# The functional *Mi-2/Foxo* complex targets *PGRP-SC2* for the *Drosophila* immune defense against bacterial infection

**DOI:** 10.3389/fimmu.2025.1664564

**Published:** 2025-09-29

**Authors:** Xianrui Zheng, Umar Ali, Yiheng Jin, Erwen Ding, Yangyang Zhu, Muhammad Usama, Qingshuang Cai, Shanming Ji

**Affiliations:** ^1^ Zhangzhou Affiliated Hospital of Fujian Medical University, Zhangzhou, Fujian, China; ^2^ Center for Developmental Biology, School of Life Sciences, Anhui Agricultural University, Hefei, Anhui, China; ^3^ Anhui Provincial Key Laboratory of Tumor Evolution and Intelligent Diagnosis and Treatment, Bengbu Medical University, Bengbu, Anhui, China

**Keywords:** Mi-2, Foxo, *PGRP-SC2*, IMD signaling pathway, antibacterial immune defense, *Drosophila melanogaster*

## Abstract

Innate immunity is orchestrated by an array of conserved signaling pathways and transcriptional regulators. While Forkhead box O (Foxo) has emerged as a pivotal transcription factor in regulating immune homeostasis, its interaction with chromatin remodeling machinery remains poorly defined. Here, we identify the chromatin remodeler Mi-2 as a crucial component of the *Drosophila* antibacterial immune defense. Silencing of *Mi-2* abrogates the induction of antimicrobial peptides in adult flies and leads to reduced host survival following systemic bacterial challenge. Co-immunoprecipitation assays demonstrate a physical interaction between endogenous Mi-2 and Foxo in the *Drosophila* fat body. Of interest, *Foxo* silencing phenocopies *Mi-2* knockdown, suggesting a functional interdependence between the two factors. Mechanistically, the Mi-2/Foxo functional complex binds to the 5’ flanking region of *Peptidoglycan recognition protein SC2* (*PGRP-SC2*), a negative regulator of the immune deficiency (IMD) signaling pathway, to prevent *PGRP-SC2* expression. Genetic epistasis experiments support a hierarchical relationship, with *PGRP-SC2* acting downstream of *Mi-2*/*Foxo*. Collectively, our findings uncover a previously uncharacterized chromatin-based regulatory mechanism whereby Mi-2 collaborates with Foxo to mediate the antibacterial immune response in *Drosophila*.

## 1 Introduction

Innate immunity serves as the first line of the host defense against invading pathogens across metazoan species (1–4). In recent decades, *Drosophila melanogaster* (fruit fly) has been extensively utilized as a powerful animal model for dissecting the molecular mechanisms of innate immunity due to its clear genetic tractability, short life cycle, and the evolutionary conservation of key immune pathways (5, 6). Insights gained from *Drosophila* studies have significantly advanced our understanding of how innate immune responses are precisely regulated. In *Drosophila*, two major signaling pathways govern the host systemic immune response: the Toll and the immune deficiency (IMD) signaling pathways (5, 7–9). The activation of these pathways leads to the induction of a repertoire of antimicrobial peptides (AMPs), which are primarily synthesized in the fat body, the fly analog of the mammalian liver. These AMPs serve as potent immune effectors that target microbial membranes, thereby directly limiting pathogen proliferation (5, 7, 10–12).

The regulation of AMP gene expression has been extensively investigated at the level of signaling cascade and transcription factor activity (9, 11). The Toll and IMD signaling pathways initiate distinct but partially overlapping immune responses (9, 13, 14). The Toll pathway is primarily activated upon recognition of lysine-type peptidoglycans (PGNs) and fungal β-glucans by pattern recognition receptors (PRRs) such as Peptidoglycan recognition protein SA (PGRP-SA), Gram-negative bacteria binding protein 1 (GNBP1), and Peptidoglycan recognition protein SD (PGRP-SD) (15–17). This triggers a proteolytic cascade that cleaves and activates the cytokine-like molecule Spätzle (Spz), which in turn binds and activates the Toll receptor. Upon Toll activation, the adaptor proteins Myeloid differentiation factor 88 (Myd88), Tube (Tub), and the kinase Pelle (Pll) are recruited, leading to the degradation of the inhibitor Cactus (Cact) and the subsequent nuclear translocation of nucleic factor kappa B (NF-κB) transcription factors Dorsal (Dl) and Dorsal-related immunity factor (Dif). These transcription factors drive the expression of Toll-dependent AMP genes such as *Drosomycin* (*Drs*) and *Metchnikowin* (*Mtk*) (9, 18–21).

In contrast, the IMD signaling pathway is primarily activated upon detection of meso-diaminopimelic acid-type PGNs, which are characteristic of Gram-negative bacteria and some types of Gram-positive bacteria. This recognition is mediated by membrane-bound PRRs such as Peptidoglycan recognition protein LC (PGRP-LC) and intracellular receptors like Peptidoglycan recognition protein LE (PGRP-LE) (22–25). Activation of the IMD pathway results in recruitment of the adaptor protein Imd, the Fas-associated death domain (Fadd), and the caspase death related ced-3/Nedd2-like caspase (Dredd). This leads to a relatively complicated but fine-tuned signal transduction reaction by a series of modulators, and finally the cleavage and activation of the NF-κB transcription factor Relish (Rel), which enters the cell nucleus to promote transcription of IMD-dependent AMPs, including *Attacin* (*Att*), *Cecropin* (*Cec*), and *Diptericin* (*Dpt*) (9, 26–28).

Beyond canonical signaling, a growing body of evidence highlights the importance of chromatin dynamics and epigenetic mechanisms in controlling the accessibility and responsiveness of immune gene loci. These include histone modifications, nucleosome remodeling, and interactions with non-coding RNAs, all of which modulate the transcriptional landscape in response to infection (29–34). Chromatin remodeling proteins, such as members of the SWItch/sucrose nonfermentable (SWI/SNF) complex, the nucleosome remodeling and deacetylase (NuRD) complex, and the imitation switch (ISWI) complex, have emerged as pivotal regulators of transcriptional plasticity in development, differentiation, and immunity (35–40). Among these, Mi-2 is a central ATPase subunit of the NuRD complex, which coordinates ATP-dependent nucleosome remodeling with histone deacetylation to mediate gene repression (41–44). In *Drosophila*, Mi-2 has been shown to regulate embryogenesis, neuronal development and stem cell proliferation (45–48), but its role in the host immune response remains poorly characterized.

Forkhead box O (Foxo) transcription factors act as central hubs in integrating environmental cues such as nutrient status, oxidative stress, and infection (49–52). In *Drosophila*, Foxo translocates into the cell nucleus under low insulin or stress conditions and regulates the expression of genes involved in autophagy, metabolism, longevity, and immunity (53–58). Notably, Foxo has been implicated in modulating basal immune tone, maintaining gut epithelial homeostasis, and limiting systemic inflammation (56, 59). However, transcriptional activation and repression by Foxo require cooperation with chromatin-modifying enzymes and remodeling complexes, which remain largely undefined in immune contexts.

In this study, we investigate the functional relationship between Mi-2 and Foxo in the context of antibacterial immune defense in *Drosophila*. Through a combination of genetic manipulation and transcriptional profiling, we demonstrate that Mi-2 is indispensable for AMP gene induction and host survival following bacterial infection. We further show that Mi-2 physically associates with Foxo and that together, they repress the expression of *Peptidoglycan recognition protein SC2* (*PGRP-SC2*), a negative regulator of the IMD signaling pathway (59–63). Our findings reveal a novel chromatin-based mechanism through which Mi-2 and Foxo coordinate transcriptional responses to bacterial infection and provide new insights into the integration of chromatin remodeling and immune gene regulation.

## 2 Materials and methods

### 2.1 *Drosophila* strains and husbandry

Flies were raised on the standard *Drosophila* medium (6.65% cornmeal, 7.15% dextrose, 5% yeast, 0.66% agar, 2.2% nipagin, and 3.4 mL/L propionic acid) at 25°C with 60% relative humidity under a 12 h/12 h light-dark cycle. To generate specific gene silencing at the adult stage using Gal4/Gal80^ts^ system, crossings were first performed at 18°C. After eclosion, progenies were shifted to 29°C for 7 d. The following fly strains were purchased from public *Drosophila* stock centers: *Mi-2 RNAi #1* (Vienna *Drosophila* RNAi Center, #107204), *Mi-2 RNAi #2* (Bloomington *Drosophila* Stock Center, #51774), *Foxo RNAi #1* (Vienna *Drosophila* RNAi Center, #106097), *Foxo RNAi #2* (Vienna *Drosophila* RNAi Center, #107786), and *PGRP-SC2 RNAi* (Vienna *Drosophila* RNAi Center, #104578). The *lpp-Gal4*, *tub-Gal80^ts^*, *GFP RNAi*, and *w^1118^* flies were described previously (64–67).

### 2.2 Antibodies

The following primary antibodies were used in this study: mouse anti-β-Tubulin (1:3000, Cowin, Cat#CW0098M), mouse anti-Flag (1:2000, Merck, Cat#F3165), rabbit anti-Flag (1:1000, Merck, Cat#F7425), rabbit anti-Myc (1:3000, Medical & Biological Laboratories, Cat#562), rabbit anti-PGRP-SC2 (1:1000, MyBioSource, Cat#MBS9013948), rabbit anti-Foxo (1:1000, Abcam, Cat#ab195977), and rat anti-Mi-2 (1:1000, Thermo Fisher, Cat#61463). The secondary antibodies used in this study include goat anti-mouse IgG H & L (1:5000, Abcam, Cat#ab150078), goat anti-rabbit IgG H & L (1:5000, Abcam, Cat#ab6789), and goat anti-rat IgG H & L (1:5000, Abcam, Cat#ab182018).

### 2.3 Bacterial infection, fly survival, and bacterial burden assays

Bacterial cultures were grown overnight at 30°C. Cultures were then pelleted and resuspended in sterile phosphate-buffered saline (PBS) solution until the OD_600_ reached around 1. Male adult flies (3-d-old) were anesthetized with carbon dioxide on a flypad and injected with bacteria (4.6 nL) by using a tungsten nanoinjector. Subsequently, flies were carefully transferred into fresh vials (around 50 individuals per vial). Control flies were injected with the same volume of PBS solution. The detailed information of *Pectobacterium carotovorum carotovorum 15* (*Ecc15*), *Serratia marcescens* (*S. marcescens*), and *Enterococcus faecalis* (*E. faecalis*) was described previously (68, 69).

For fly survival analysis, infected flies were scored for daily mortality. Flies (< 5%) that died within 2 h post-injection were not considered. Survival data were collected from 3 biological replicates and shown as means plus standard errors.

For bacterial burden assays, flies (10 individuals for each sample) were homogenized in sterile PBS buffer, followed by serial dilutions, and finally, 100 μL of each diluent was spread on a Luria Bertani (LB) agar plate. All LB plates were further incubated at 30°C for 24 h. Flies that were collected immediately after bacterial injection were put in the 0-d group. The number of bacterial colonies was counted, and data were pooled from 21 independent biological replicates.

### 2.4 RT-qPCR

Reverse transcription plus quantitative polymerase chain reaction (RT-qPCR) experiments were performed according to a previously described protocol (70). In brief, total RNA was extracted from dissected fat body tissues or whole flies using TRIzol reagent (Thermo Fisher, Cat#15596026). cDNA synthesis was performed using the TransScript All-in-one First-Strand cDNA Synthesis SuperMix kit (TransGen, Cat#AT341-01). Quantitative PCR was carried out using the SYBR Green One-Step kit (TransGen, Cat#AQ211-01) on a Light Cycler 480, in which *RpL32* was used as an endogenous control. Relative fold changes were calculated using the ΔΔCt method. Data were collected from 5 independent biological replicates. The detailed information of gene-specific primers used in RT-qPCR is shown in **Supplementary Table S1**.

### 2.5 Western blotting

Whole flies or dissected fat body tissues were lysed in lysis buffer (150 mM NaCl, 50 mM Tris-HCl, pH = 7.5, 10% glycerol, 0.5% Triton X-100, and 1 mM PMSF). Samples were centrifuged at 13,000 rpm at 4°C for 30 min. The supernatant was collected and resolved on a 10% SDS-PAGE gel, transferred to a PVDF membrane, and probed with primary antibodies at 4°C overnight. After incubation with secondary antibodies for 1 h at room temperature, the membrane was subjected to Western blot assay by using the enhanced chemiluminescence substrate.

### 2.6 Co-IP

S2 cells were cultured in the insect medium supplemented with 10% fetal bovine serum and transfected with indicated expression plasmids. After 48 h, cells were lysed in lysis buffer (150 mM NaCl, 50 mM Tris-HCl, pH = 7.5, 10% glycerol, 0.5% Triton X-100, and 1 mM PMSF). For *in vivo* samples, the fat body tissues were dissected from *w^1118^* flies, and lysates were prepared as described above. After centrifugation (12,000 rpm) at 4°C for 10 min, 1/10 of the supernatant was collected as the “Input” sample. The remaining supernatant was incubated with indicated antibodies and agarose beads for immunoprecipitation at 4°C overnight. Samples were then washed with wash buffer (50 mM Tris-HCl, pH = 7.5, 500 mM NaCl, 0.5% Triton X-100, and 10% glycerol) at 4°C for 3 times (1 h in total), followed by Western blot experiments. Twenty percent of the immunoprecipitant (IP) was used for the detection of immunoprecipitation efficiency, whereas eighty percent was used for co-IP examination.

### 2.7 Identification of Mi-2 interactome via IP-LC-MS/MS

The immunoprecipitation and liquid chromatography plus tandem mass spectrometry (IP-LC-MS/MS) was performed as described previously (71). Briefly, *Drosophila* S2 cells were transfected with Flag-Mi-2 expression plasmids, and immunoprecipitation was performed as described above. Flag-GFP was expressed in the control group. After immunoprecipitation, samples were washed with wash buffer (50 mM Tris-HCl, pH = 7.5, 500 mM NaCl, 0.5% Triton X-100, and 10% glycerol) at 4°C for 3 times (1 h in total), followed by incubation with Flag peptide at 4°C for 30 min. After centrifugation (12,000 rpm) at 4°C for 2 min, the supernatant was transferred into a fresh Eppendorf tube, followed by digestion with Trypsin (Thermo Fisher, Cat#90057) at 37°C for 30 min. Samples were then desalted using the Pierce™ C-18 spin column (Thermo Fisher, Cat#89870) and subjected to LC-MS/MS analysis to identify the interactome of Mi-2. The LC-MS/MS data were processed using the Thermo Proteome Discovery (version 1.4.1.14) and searched against the UniProt-*Drosophila* database. The raw data is available online (https://data.mendeley.com/preview/3xshb9x6w4?a=53354993-4775-4d61-b454-bf123a85bf89).

### 2.8 ChIP-qPCR

The chromatin immunoprecipitation plus quantitative polymerase chain reaction (ChIP-qPCR) experiments were carried out according to protocols published previously (72). In detail, fat bodies were dissected from 100 adult male flies and incubated in 10 mL ice-cold swelling buffer (0.1 M Tris-HCl, pH = 7.5, 10 mM KOAc, 15 mM MgOAc, 1% NP-40, and 1 mM PMSF). Samples were homogenized for 2 min using a loose-fitting Dounce homogenizer, fixed with 1% formaldehyde for 10 min, and quenched with 125 mM glycine to stop fixation. After centrifugation at 1000 g for 5 min at 4°C, the pellet was resuspended in 10 mL fresh swelling buffer and filtered through 70 μm and 40 μm cell strainers, respectively. Samples were centrifuged at 1000 g for 5 min to obtain the nuclear pellet, followed by nuclear lysate preparation by using lysis buffer (50 mM Tris-HCl, pH = 7.5, 10 mM EDTA, 1% SDS, 1 mM DTT, and 1 mM PMSF). Samples were then sonicated for 30 min at 4°C. Immunoprecipitation was performed using anti-Mi-2 or anti-Foxo antibodies. Enrichment at different regions of *PGRP-SC2* was assessed by qPCR using specific primers (**Supplementary Table S1**).

### 2.9 Statistical analysis

Statistical analyses were conducted using GraphPad Prism (version 10.1.2.324). The one-way ANOVA followed by Tukey’s *post hoc* test was applied where appropriate. Survival curves were compared using the Log-Rank test. The *P* < 0.05 was considered statistically significant. *, *P* < 0.05; **, *P* < 0.01; ***, *P* < 0.001; ns, not significant.

## 3 Results

### 3.1 *Drosophila Mi-2* is essential for the induction of antimicrobial peptides in response to bacterial infection

**Figure 1:**
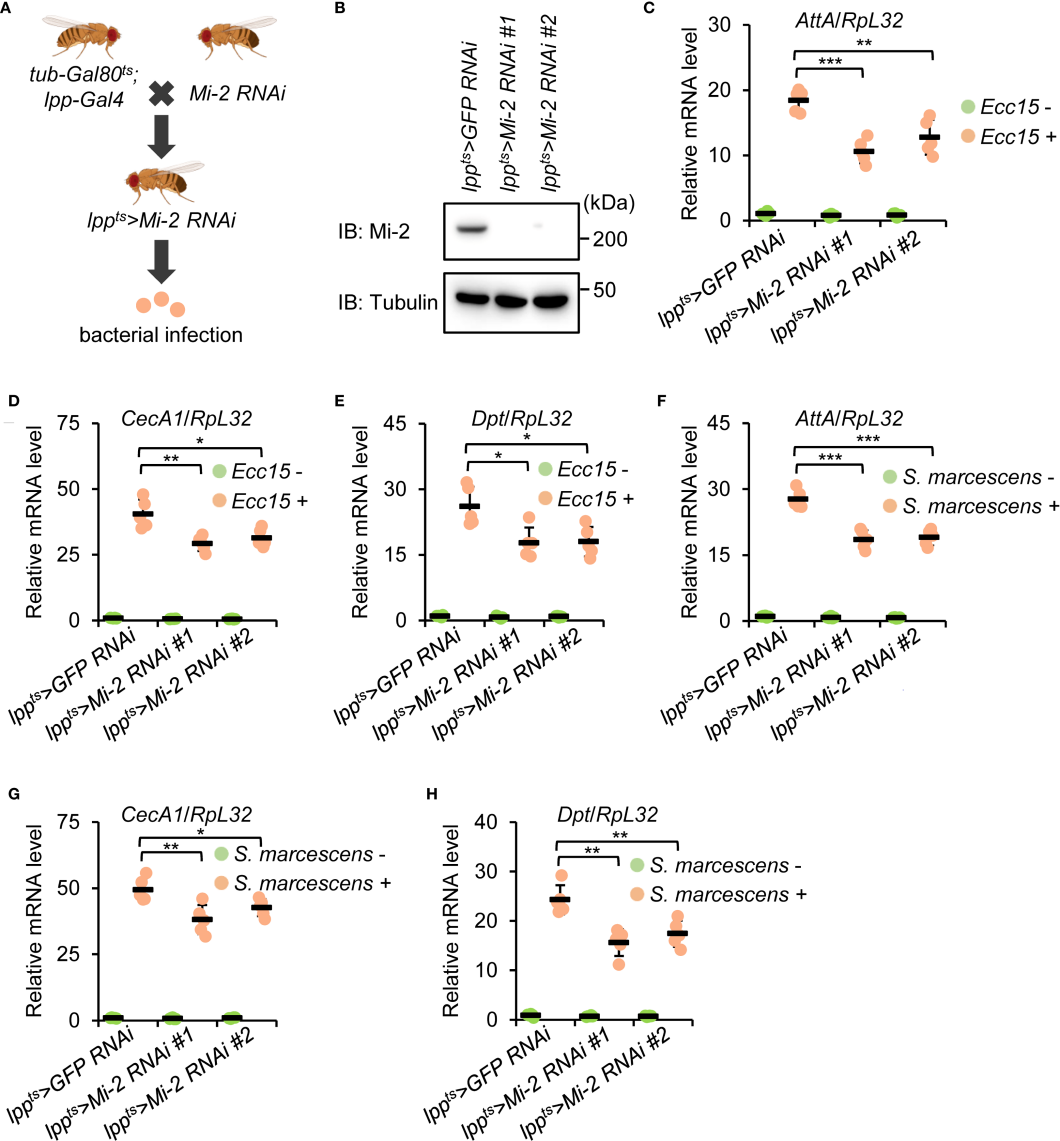
*Drosophila Mi-2* is required for AMP induction following bacterial infection. **(A)** The diagram showing genetic manipulations to obtain flies with the fat body-specific silencing of *Mi-2*. **(B)** Western blot monitoring Mi-2 protein levels in the fat body dissected from control and *Mi-2 RNAi* flies. Tubulin was used as the loading control. **(C-E)** Adult flies, including *lpp^ts^>GFP RNAi* (control), *lpp^ts^>Mi-2 RNAi #1*, and *lpp^ts^>Mi-2 RNAi #2*, were infected with *Ecc15*, followed by RT-qPCR assays to examine the transcript levels of *AttA* **(C)**, *CecA1* **(D)**, and *Dpt* **(E)**. **(F-H)** Similar RT-qPCR experiments were performed as in C-E, except that *S. marcescens* were used for infection. In C-H, data were collected from 5 independent replicates and shown as means plus standard errors. *P < 0.05; **P < 0.01; ***P < 0.001.

The loss-of-function mutant flies of *Mi-2* are not viable due to severe defects in early embryonic development (47, 73). To assess the potential involvement of *Mi-2* in the *Drosophila* innate immune response, we silenced *Mi-2* specifically in the fat body using the Gal4/UAS system (*lpp-Gal4* driver). In addition, we utilized the *tub-Gal80^ts^* strain to drive *Mi-2* silencing (referred to as *lpp^ts^>Mi-2 RNAi #1* and *lpp^ts^>Mi-2 RNAi #2*) at the adult stage (**Figure 1A**). Western blot experiments confirmed the knockdown efficiency of the two different *Mi-2 RNAi* lines in the *Drosophila* fat body (**Figure 1B**). We further challenged these flies and the age-paired controls (*lpp^ts^>GFP RNAi*) with *Pectobacterium carotovorum carotovorum 15* (*Ecc15*). *Ecc15* is one type of Gram-negative bacterial pathogens activating the immune deficiency (IMD) pathway in *Drosophila* (74). Quantitative reverse transcription plus polymerase chain reaction (RT-qPCR) analyses revealed that the induction of AMPs downstream of IMD signaling, including *Attacin A* (*AttA*), *Cecropin A1* (*CecA1*), and *Diptericin* (*Dpt*), was impaired in *Mi-2* knockdown flies compared to those in controls (**Figures 1C–E**). These data indicate that *Mi-2* is required for robust AMP gene expression in response to bacterial infection. Consistently, we observed decreased transcript levels of *AttA*, *CecA1*, and *Dpt* in *Mi-2 RNAi* flies when we used *Serratia marcescens* (*S. marcescens*), another type of Gram-negative bacterial pathogens, for infection treatment (**Figures 1F-H**).

### 3.2 Silencing of *Mi-2* compromises the *Drosophila* survival and bacterial clearance activity

**Figure 2:**
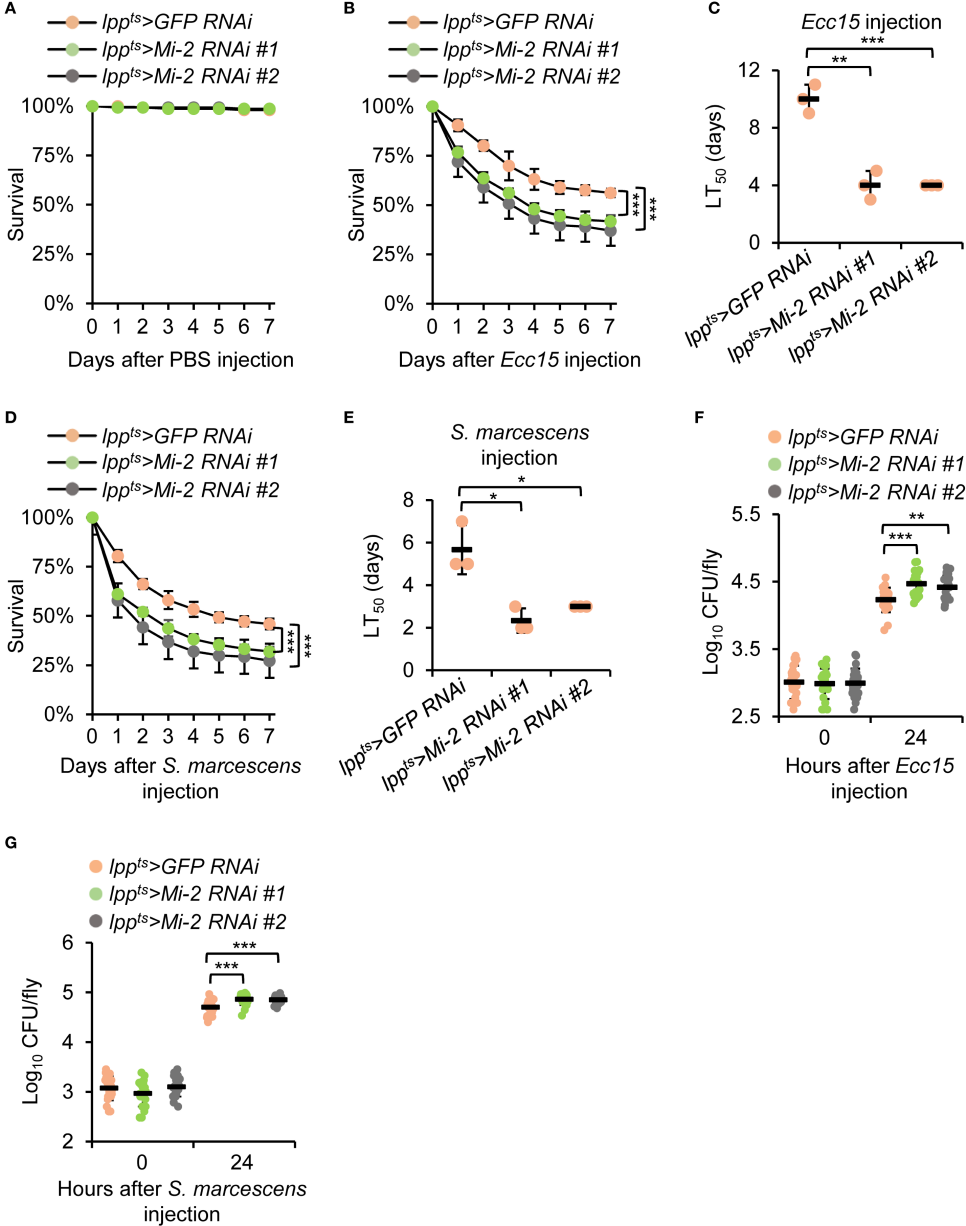
*Mi-2* is essential for the *Drosophila* defense against bacterial challenge. **(A-C)** Survival curves of *Mi-2 RNAi* and control flies after the injection of PBS **(A)** or *Ecc15* **(B)**. The number of flies is as follows. In A, *lpp^ts^>GFP RNAi*: 50, 48, 50; *lpp^ts^>Mi-2 RNAi #1*: 49, 49, 50; *lpp^ts^>Mi-2 RNAi #2*: 50, 50, 49. In B, *lpp^ts^>GFP RNAi*: 50, 48, 48; *lpp^ts^>Mi-2 RNAi #1*: 49, 49, 48; *lpp^ts^>Mi-2 RNAi #2*: 49, 47, 50. The time points when half of the experimental flies **(B)** died (referred to as LT_50_) are shown in **(C–E)** Survival assays were performed as in A-C, except that *S. marcescens* were used for injection. In **(D)**, the number of flies is as follows. *lpp^ts^>GFP RNAi*: 49, 50, 49; *lpp^ts^>Mi-2 RNAi #1*: 48, 49, 50; *lpp^ts^>Mi-2 RNAi #2*: 48, 48, 48. (**F, G**) Bacterial load (CFU per fly) at 24 h post-infection with *Ecc15* **(F)** *or S. marcescens* **(G)**. In **(A-E)**, data were collected from 3 independent replicates and shown as means plus standard errors. In **(F, G)**, data were pooled from 21 independent replicates. *P < 0.05; **P < 0.01; ***P < 0.001.

To determine the physiological relevance of *Mi-2* in the *Drosophila* antibacterial immune defense, we performed survival assays following bacterial infections. *Mi-2* knockdown flies exhibited a reduction in survival compared to controls after *Ecc15* injection while they survived in a similar way after the injection of sterile phosphate-buffered saline (PBS) solution (**Figures 2A, B**). The median survival time of *Mi-2 RNAi* flies after *Ecc15* injection was decreased by more than 50% (**Figure 2C**). A similar trend was observed for *S. marcescens* injection, where *Mi-2*-silenced flies showed a median survival of 2.3 d, compared to 5.7 d in controls (**Figures 2D, E**).

To assess the fly efficiency of bacterial clearance, we measured colony-forming units (CFUs) in whole-fly homogenates at 24 h post-infection of *Ecc15* or *S. marcescens*. *Mi-2* knockdown flies displayed higher bacterial loads than control flies (**Figures 2F, G**). These findings demonstrate that *Mi-2* is essential for *Drosophila* survival and effective bacterial elimination upon infection.

### 3.3 *Mi-2* is dispensable for mediating the *Drosophila* Toll antibacterial immune defense

To determine whether *Drosophila Mi-2* is also involved in the Toll pathway-mediated immune response, we assessed the expression of Toll-dependent AMPs and host survival following Gram-positive bacterial infection. Adult flies with fat body-specific *Mi-2* knockdown were challenged with *Enterococcus faecalis* (*E. faecalis*), a bacterial pathogen known to activate the Toll signaling pathway in *Drosophila* (74). RT-qPCR analyses revealed no significant differences in the *E. faecalis*-driven induction of *Drosomycin* (*Drs*) and *Metchnikowin* (*Mtk*) between *Mi-2 RNAi* and control flies (**Supplementary Figures S1A, B**). Consistently, survival assays showed that *Mi-2* knockdown flies exhibited comparable resistance to *E. faecalis* infection as control flies (**Supplementary Figure S1C**). Bacterial burden analyses displayed similar *E. faecalis* proliferation levels between *Mi-2 RNAi* and control flies (**Supplementary Figure S1D**). These results collectively indicate that *Mi-2* is not required for the Toll-mediated antimicrobial response, and its function in *Drosophila* innate immunity is specific to the IMD pathway.

### 3.4 Mi-2 physically interacts with Foxo

**Figure 3:**
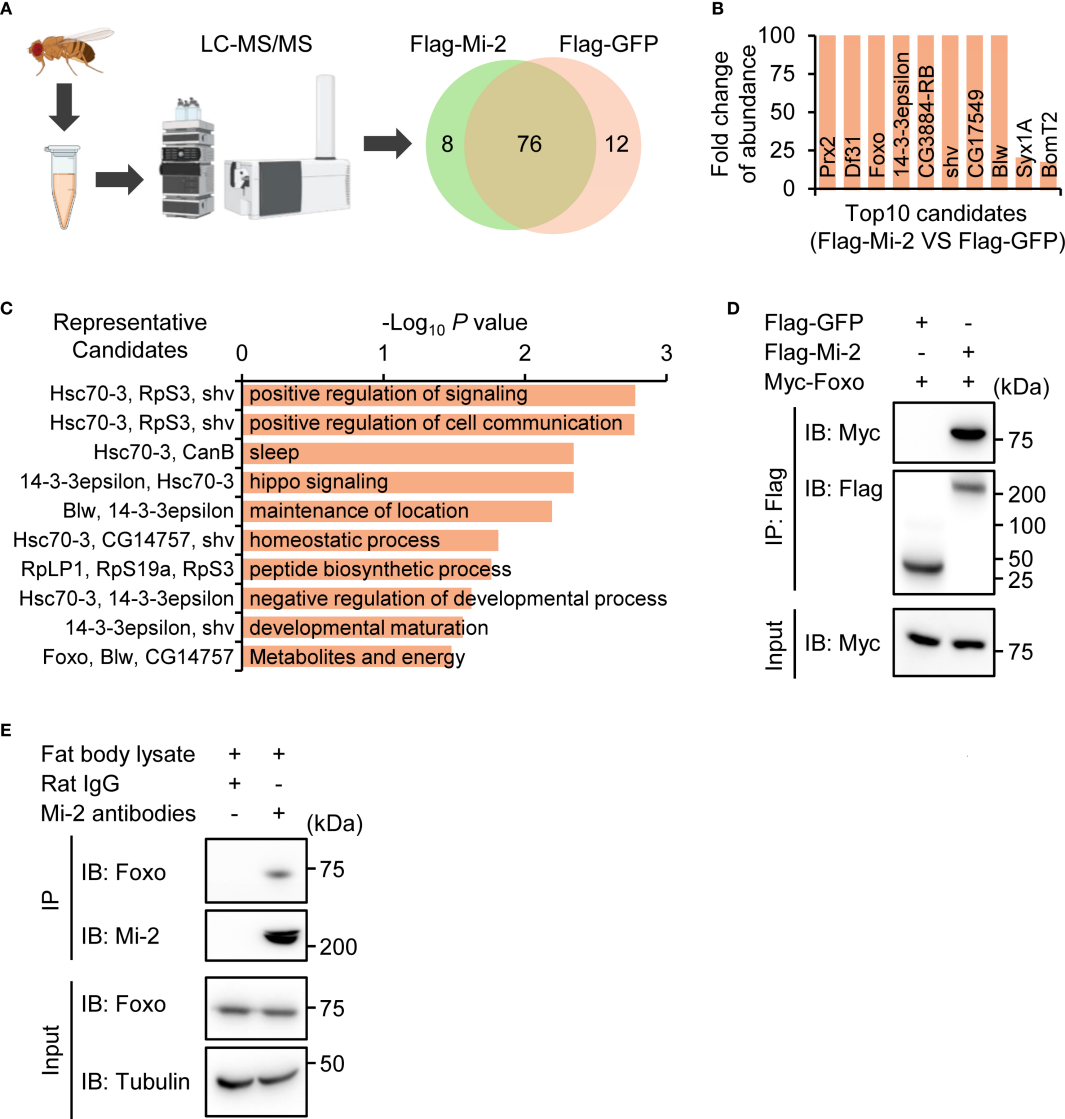
Mi-2 physically interacts with Foxo. **(A)** Schematic of IP-LC-MS/MS to identify potential Mi-2 interacting protein candidates. **(B)** The top 10 candidates of the Mi-2 interactome. **(C)** GO analysis of the Mi-2 interactome. **(D)** Co-IP from S2 cells expressing Flag-Mi-2 and Myc-Foxo. Input and IP blots are shown for Flag or Myc. **(E)** The fat body tissues were dissected from *w^1118^* flies, followed by co-IP assays using anti-Mi-2 antibodies. Rat IgG was used in the control sample.

We explored the molecular mechanism by which Mi-2 modulates the *Drosophila* IMD antibacterial immune defense. For this, we transfected cultured *Drosophila* S2 cells with plasmids expressing Flag-tagged Mi-2. By performing immunoprecipitation and liquid chromatography plus tandem mass spectrometry (IP-LC-MS/MS) experiments (**Figure 3A**), we identified 28 proteins/peptides that potentially interact with Mi-2 (**Figure 3B**, **Supplementary Table S2**). Gene ontology (GO) analyses of these Mi-2-associated candidates revealed that they predominantly belonged to categories, including signaling homeostasis, cell communication, and sleep (**Figure 3C**). Intriguingly, we noted one candidate, Forkhead box O (Foxo), which was previously reported to physically associate with Mi-2 (75). Given that *Drosophila* Foxo has been implicated in regulating immune gene expression (59, 76, 77) and that chromatin remodeling complexes often interact with sequence-specific transcription factors, we hypothesized that Mi-2 may form a functional complex with Foxo for immune regulation in *Drosophila*. To test this idea, we co-transfected *Drosophila* S2 cells with Flag-tagged Mi-2 and Myc-tagged Foxo constructs and performed co-immunoprecipitation (co-IP) assays. Foxo was specifically pulled down by anti-Flag beads only in the presence of Mi-2 (**Figure 3D**), confirming their physical interaction. To explore whether Mi-2 forms a functional complex with Foxo *in vivo*, we dissected the *Drosophila* fat body tissue for co-IP experiments using anti-Mi-2 antibodies. Our results indicated that the endogenous Mi-2 and Foxo associate with each other in the *Drosophila* fat body (**Figure 3E**).

### 3.5 *Foxo RNAi* phenocopies *Mi-2 RNAi* in regulating *Drosophila* innate immunity

**Figure 4:**
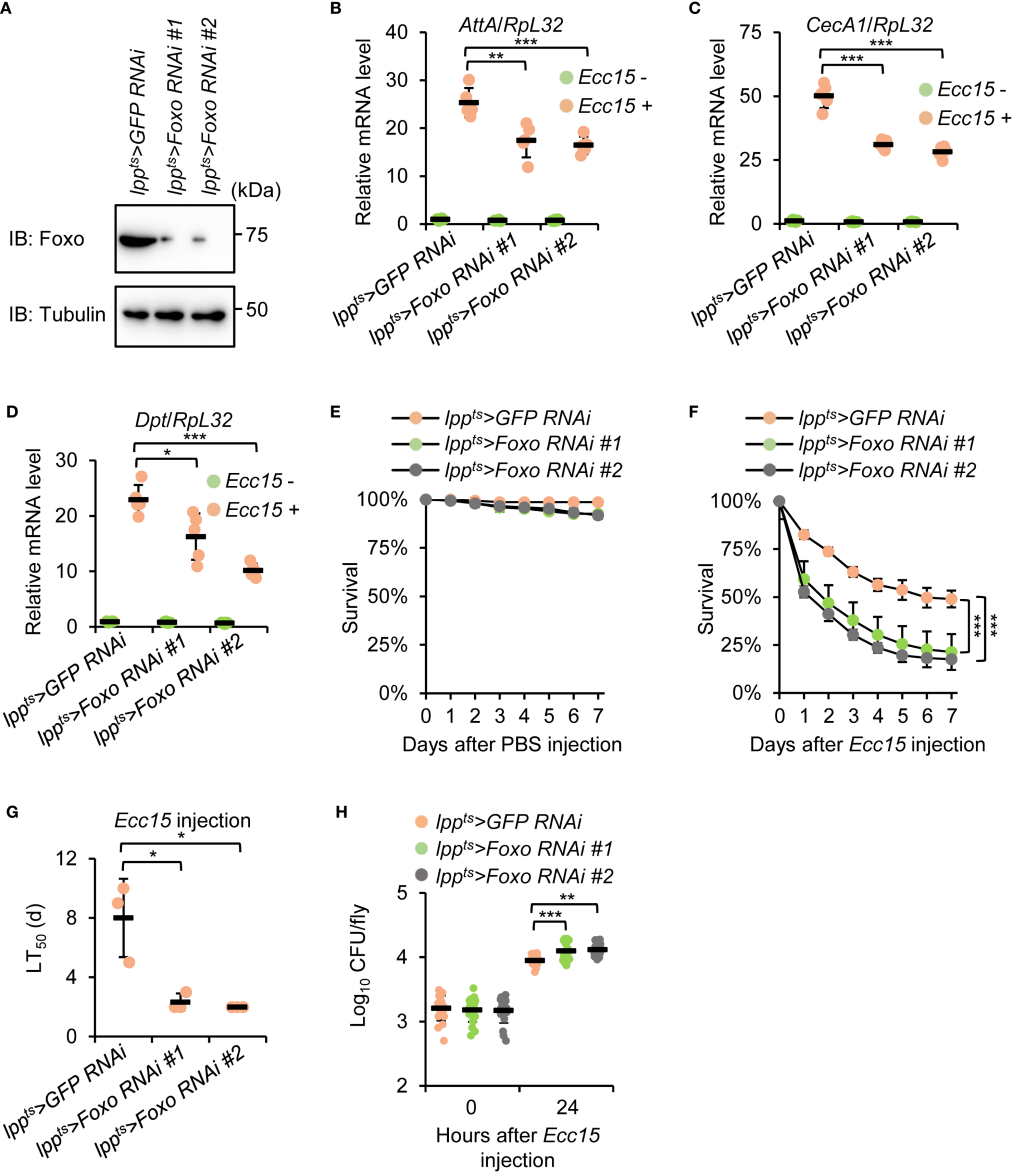
*Foxo* knockdown phenocopies *Mi-2* depletion in the *Drosophila* antibacterial immune defense. **(A)** Western blots showing the knockdown efficiency of different *Foxo RNAi* strains. Tubulin was used as the loading control. **(B-D)** RT-qPCR of AMP genes in fat body-specific *Foxo RNAi* flies after *Ecc15* infection. **(E-G)** Survival assays of *Foxo RNAi* and control flies after *Ecc15* challenge. The number of flies is as follows. In E, *lpp^ts^>GFP RNAi*: 50, 48, 49; *lpp^ts^>Foxo RNAi #1*: 50, 49, 50; *lpp^ts^>Foxo RNAi #2*: 49, 49, 49. In F, *lpp^ts^>GFP RNAi*: 49, 50, 50; *lpp^ts^>Foxo RNAi #1*: 48, 48, 49; *lpp^ts^>Foxo RNAi #2*: 50, 48, 50. The time points when half of the experimental flies **(F)** died (LT_50_) were shown in **(G, H)** Bacterial load assays at 24 h after *Ecc15* infection. In **(B–D)**, data were collected from 5 independent replicates and shown as means plus standard errors. In **(E–G)**, data were collected from 3 independent replicates. In **(H)**, data were pooled from 21 independent replicates. *P < 0.05; **P < 0.01; ***P < 0.001.

To determine whether *Foxo* functions in the same genetic pathway as *Mi-2*, we first performed *Foxo* knockdown using the Gal4/UAS system as described above (**Figure 4A**). We next analyzed the immune response of these flies upon bacterial infection. Similar to *Mi-2 RNAi* flies, *Foxo*-silenced flies showed reduced expressions of *AttA*, *CecA1*, and *Dpt* following *Ecc15* injection (**Figures 4B–D**). Furthermore, *Foxo* knockdown flies exhibited heightened susceptibility to *Ecc15* infection, with median survival reduced by around 6 d (**Figures 4E–G**). Of note, the *Ecc15* burden in *Foxo RNAi* flies was increased by more than 50%, compared to that of control flies (**Figure 4H**). These results suggest that *Mi-2* and *Foxo* function cooperatively to regulate the antibacterial immune response in *Drosophila*.

### 3.6 Mi-2 and Foxo suppress *PGRP-SC2* expression in the *Drosophila* fat body

**Figure 5:**
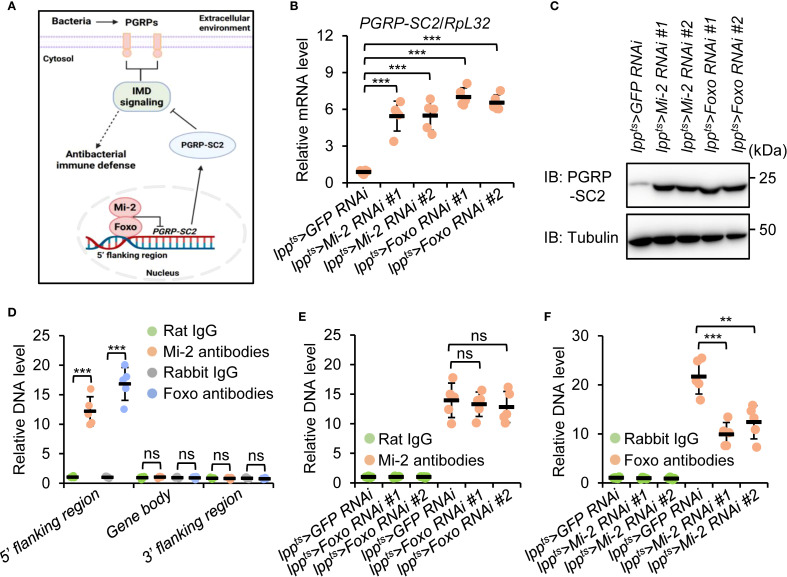
Mi-2 and Foxo antagonize *PGRP-SC2* expression in the *Drosophila* fat body. **(A)** The diagram illustrating a working model in which *Drosophila* Mi-2 forms a functional complex with Foxo and prevents the expression of PGRP-SC2, thereby ensuring a robust immune response upon bacterial challenge. **(B, C)** RT-qPCR **(B)** and Western blot **(C)** assays validating the expression levels of *PGRP-SC2* in the fat body dissected from indicated flies. **(D-F)** ChIP-qPCR experiments showing the enrichment of Mi-2 and Foxo at different regions of *PGRP-SC2*. In **(B, D–F)**, data were collected from 5 independent replicates and shown as means plus standard errors. **P < 0.01; ***P < 0.001; ns, not significant.

Previous studies have demonstrated that Foxo prevents the expression of *Peptidoglycan recognition protein SC2* (*PGRP-SC2*), which encodes a typical amidase that downregulates IMD signaling (59–63). We therefore proposed a working model in which Mi-2 forms a functional complex with Foxo to antagonize the expression of *PGRP-SC2*, thereby maintaining a robust transactivation of IMD signaling upon bacterial infection (**Figure 5A**). To test our proposal, we performed both RT-qPCR and Western blot experiments. As illustrated in **Figures 5B, C**, *PGRP-SC2* expression was elevated in both *Mi-2 RNAi* and *Foxo RNAi* flies. We further carried out chromatin immunoprecipitation plus quantitative polymerase chain reaction (ChIP-qPCR) assays and found that both Mi-2 and Foxo were enriched at the 5’ flanking region of *PGRP-SC2* (**Figure 5D**), suggesting a direct transcriptional repression of *PGRP-SC2* by the Mi-2/Foxo complex.

To illustrate how Mi-2/Foxo bind to the 5’ flanking region of *PGRP-SC2*, we performed ChIP-qPCR assays in either *Mi-2 RNAi* or *Foxo RNAi* flies. Silencing of *Foxo* didn’t affect the binding of Mi-2 to the *PGRP-SC2* 5’ flanking region (**Figure 5E**). However, knockdown of *Mi-2* prevented the existence of Foxo at the *PGRP-SC2* 5’ flanking region (**Figure 5E**). Taken together, our data indicate that Foxo binds to the 5’ flanking region of *PGRP-SC2* and represses *PGRP-SC2* expression in a Mi-2-dependent manner.

### 3.7 Genetic epistasis places *PGRP-SC2* downstream of Mi-2/Foxo

**Figure 6:**
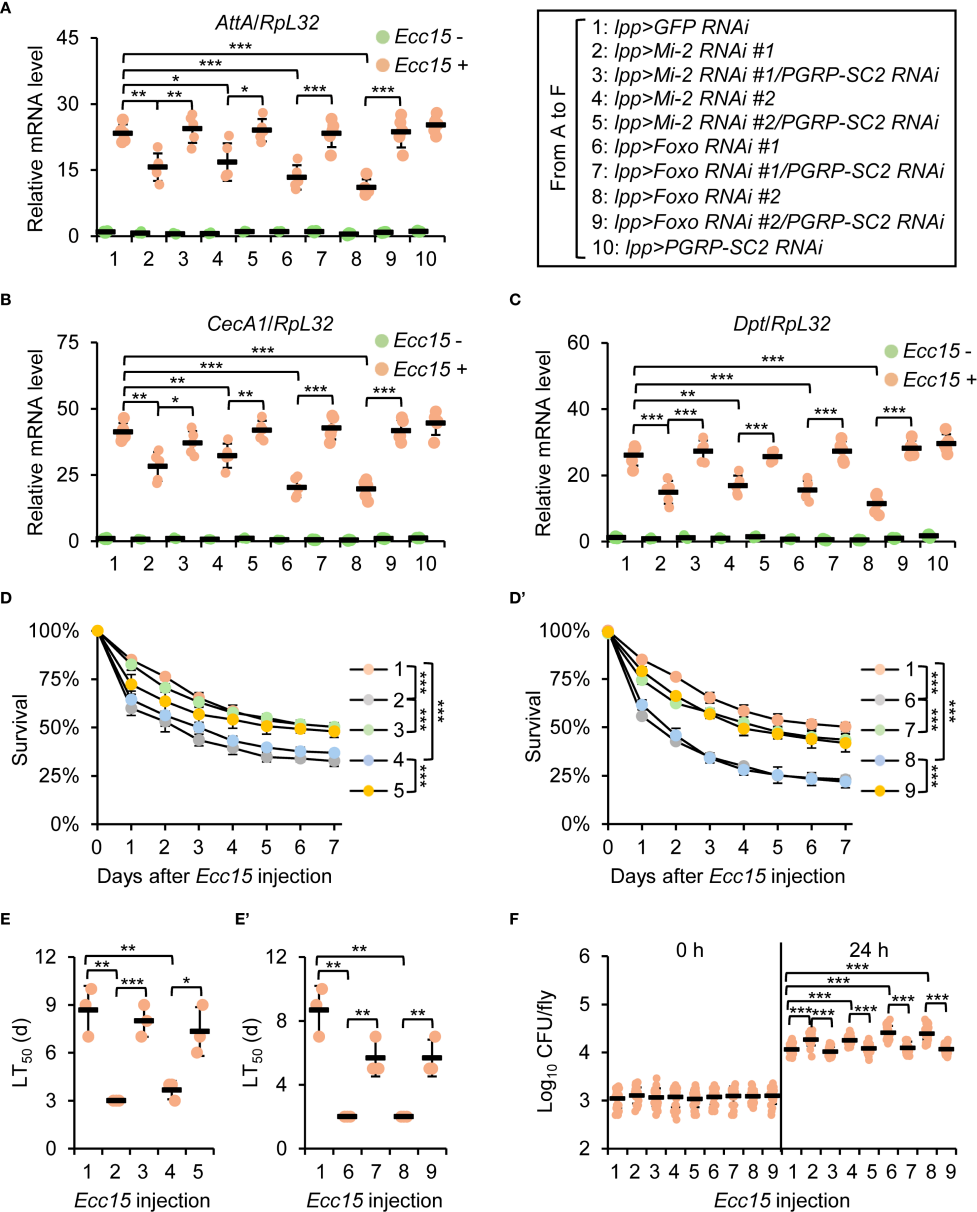
Genetic interaction between Mi-2/Foxo and *PGRP-SC2*. **(A-C)** RT-qPCR of AMPs in double knockdown of *Mi-2*;*PGRP-SC2* or *Foxo;PGRP-SC2*, compared to single knockdown. **(D**-E’**)** Survival curves comparing *Mi-2 RNAi*, *Foxo RNAi*, and double RNAi flies. The number of flies is as follows. In **(D)**, *lpp>GFP RNAi*: 49, 48, 50; *lpp>Mi-2 RNAi #1*: 48, 50, 49; *lpp>Mi-2 RNAi #1;PGRP-SC2 RNAi*: 49, 50, 50; *lpp>Mi-2 RNAi #2*: 48, 48, 50; *lpp>Mi-2 RNAi #2;PGRP-SC2 RNAi*: 49, 50, 49. In D’, *lpp>GFP RNAi*: 49, 48, 50; *lpp>Foxo RNAi #1*: 50, 49, 48; *lpp>Foxo RNAi #1;PGRP-SC2 RNAi*: 49, 48, 50; *lpp>Foxo RNAi #2*: 50, 48, 49; *lpp>Foxo RNAi #2;PGRP-SC2 RNAi*: 50, 49, 48. The time points when half of the experimental flies (**D**, D’**)** died (LT_50_) are shown in **(E**, E’**)**, respectively. **(F)** Bacterial load assays in indicated flies. In **(A–C)**, data were collected from 5 independent replicates (10 flies for each replicate) and shown as means plus standard errors. In **(D**–E’**)**, data were collected from 3 independent replicates. In **(F)**, data were pooled from 21 independent replicates (10 flies for each replicate). *P < 0.05; **P < 0.01; ***P < 0.001.

To functionally validate the role of *PGRP-SC2* as a downstream target of the Mi-2/Foxo complex, we performed genetic interaction experiments. Double knockdown of *Mi-2* and *PGRP-SC2* rescued AMP expression and fly survival compared to *Mi-2* knockdown alone (**Figures 6A–E**, **S2A**). Bacterial load was also markedly reduced in double knockdown flies (**Figure 6F**). In addition, similar results were obtained by using *Foxo* and *PGRP-SC2* double RNAi flies (**Figures 6A–F** and **S2B**). These results support a model wherein Mi-2 and Foxo cooperatively repress *PGRP-SC2* to promote effective immune activation in the fly defense against bacterial infection.

## 4 Discussion

This study identifies a previously unrecognized function of the chromatin remodeler Mi-2 in the innate immune defense of *Drosophila melanogaster* and establishes a mechanistic partnership with the transcription factor Foxo. Our results provide compelling evidence that Mi-2 is indispensable for the effective induction of AMPs and protection against bacterial infection in *Drosophila*. Moreover, the discovery of physical interaction between Mi-2 and Foxo advances our understanding of chromatin-level regulation of immune responses.

A central finding of this work is the convergence of chromatin remodeling and transcription factor signaling at the level of immune regulation. Foxo, a key effector of insulin signaling and stress responses, has been shown to regulate subsets of AMPs and immune-related genes (59, 76, 77). However, its broader regulatory potential in host defense is constrained by chromatin architecture. Mi-2, as part of the NuRD complex, remodels nucleosomes and contributes to both gene repression and activation depending on context (41–44). Our data show that Mi-2 is necessary for Foxo to suppress the expression of *PGRP-SC2*, which encodes a negative regulator of the IMD signaling pathway (59–63). This suggests that chromatin accessibility and histone deacetylation events mediated by Mi-2 are required for the repressive function of Foxo. Our follow-up projects would be focusing on exploring the mechanistic details of how Mi-2 influences Foxo recruitment to the 5’ flanking region of *PGRP-SC2*, for instance the potential changes in chromatin accessibility (via ATAC-seq), the status of histone modifications (via H3K9me3 and/or H3K27ac ChIP assays), and the post-translational modifications or localization dynamics of Foxo. These are indeed important and relevant avenues of investigations that could help elucidate the molecular basis of the Mi-2/Foxo regulatory axis.

The rescue of AMP expression and survival in *Mi-2* or *Foxo* knockdown flies by co-silencing of *PGRP-SC2* highlights the regulatory hierarchy in this axis. This genetic interaction provides not only functional validation of *PGRP-SC2* as a downstream effector but also situates Mi-2/Foxo as key upstream regulators that fine-tune immune sensitivity. The functional importance of this repression in flies is particularly evident during infection, where an optimal level of immune activation is crucial. The repression of IMD signaling via *PGRP-SC2* could dampen AMP production (59–63). Therefore, the repression of *PGRP-SC2* expression through Mi-2/Foxo action promotes rapid immune mobilization.

The implications of this work extend beyond innate immunity. Foxo is a central node in the regulation of longevity, stress resistance, and metabolism (49–52). By uncovering Mi-2 as a critical cofactor, we open new avenues for understanding how chromatin remodeling integrates environmental cues and transcriptional responses. Furthermore, since excessive immune activation or chronic inflammation underlies many age-related pathologies, elucidating Mi-2/Foxo-mediated repression mechanisms may inform strategies to modulate immune tone for therapeutic benefit. Future studies should explore the dynamic recruitment of Mi-2/Foxo to target loci upon infection, the potential involvement of additional NuRD subunits, and whether similar regulatory paradigms govern other immune genes or signaling pathways. Integration with metabolomics and epigenomics could also reveal how nutrient availability or stress conditions influence Mi-2/Foxo function and chromatin landscape in the fat body and other tissues.

In summary, we demonstrate that Mi-2 and Foxo cooperate to modulate antibacterial defense in *Drosophila*, in part through the repression of *PGRP-SC2*. This chromatin-transcription interface represents a novel regulatory layer in immune homeostasis and highlights the importance of integrating chromatin remodeling with signal-dependent gene expression programs. While this study provides compelling evidence for the functional interaction between Mi-2 and Foxo in regulating *Drosophila* antibacterial immunity, it primarily focuses on one downstream target, *PGRP-SC2*, and does not explore other potential transcriptional targets of the Mi-2/Foxo complex. Additionally, the dynamic recruitment of Mi-2 and Foxo to immune loci under varying physiological or stress conditions remains uncharacterized.

## Data Availability

The datasets presented in this study can be found in online repositories. The names of the repository/repositories and accession number(s) can be found in the article/**Supplementary Material**.
