# Supplementary material for: The functional Mi-2/Foxo complex targets PGRP-SC2 for the Drosophila immune defense against bacterial infection
Source: Front Immunol. 2025 Sep 29;16:1664564. doi: 10.3389/fimmu.2025.1664564 (PMC12515809; doi:10.3389/fimmu.2025.1664564)
Supplement: Supplementary file 1 [file Table1.docx]

Supplementary Material

# 1 Supplementary Figures and Tables

## 1.1 Supplementary Figures


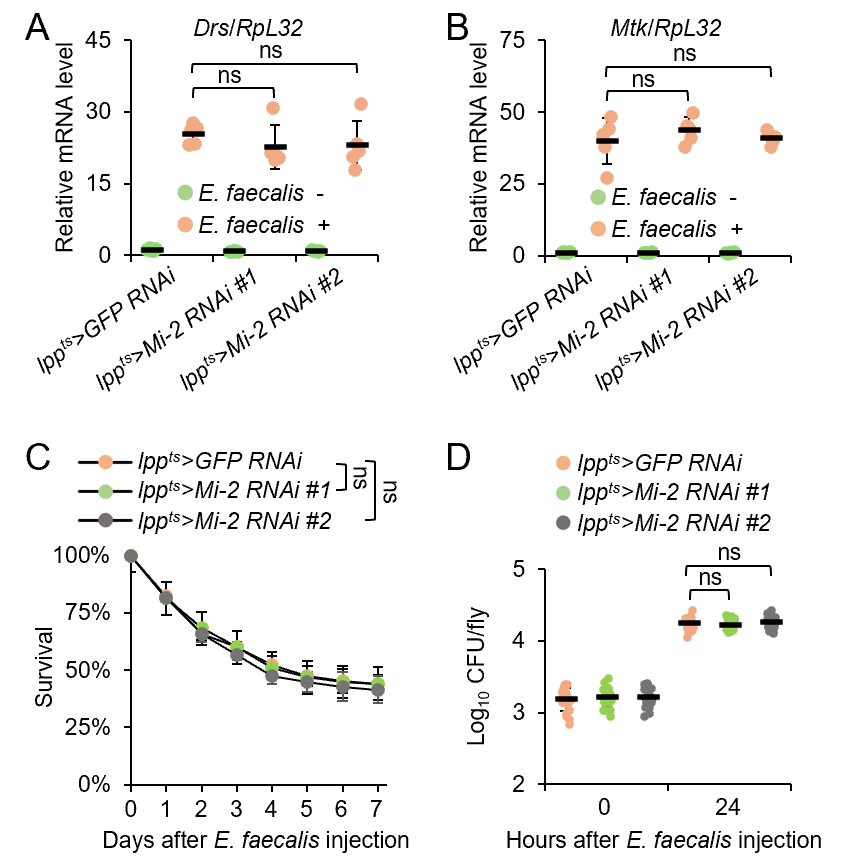


**Supplementary Figure 1.** ***Mi-2* is dispensable for modulating the *Drosophila* Toll immune defense against *E. faecalis* infection.**

(A and B) Adult flies were infected with *E. faecalis* (referred to as *E. faecalis* +) or without infection treatment (referred to as *E. faecalis* -) as indicated, followed by RT-qPCR assays to monitor the expression levels of *Drs* (A) or *Mtk* (B). (C and D) Flies were injected with *E. faecalis* as indicated, followed by survival (C) or bacterial load (D) assays. The number of flies in C is as follows. *lpp^ts^>GFP RNAi*: 48, 50, 48; *lpp^ts^>Mi-2 RNAi #1*: 48, 48, 49; *lpp^ts^>Mi-2 RNAi #1*: 49, 48, 50.


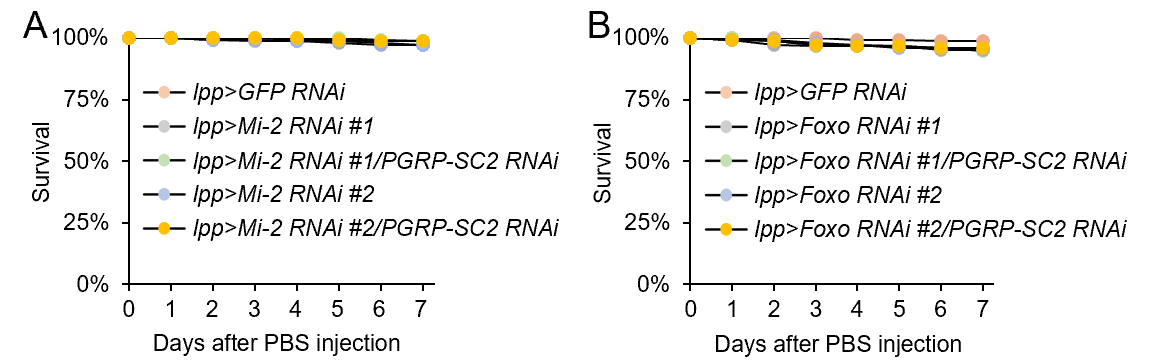


**Supplementary Figure 2. Survival curves of indicated flies after PBS injection.**

(A and B) Adult flies were injected with PBS solution as indicated, followed by survival analyses. The number of flies is as follows. In A, *lpp>GFP RNAi*: 50, 49, 49; *lpp>Mi-2 RNAi #1*: 50, 49, 50; *lpp>Mi-2 RNAi #1/PGRP-SC2 RNAi*: 50, 50, 50; *lpp>Mi-2 RNAi #2*: 49, 48, 50; *lpp>Mi-2 RNAi #2/PGRP-SC2 RNAi*: 50, 48, 49. In B, *lpp>GFP RNAi*: 50, 49, 49; *lpp>Foxo RNAi #1*: 49, 49, 50; *lpp>Foxo RNAi #1/PGRP-SC2 RNAi*: 50, 49, 49; *lpp>Foxo RNAi #2*: 48, 48, 50; *lpp>Foxo RNAi #2/PGRP-SC2 RNAi*: 50, 49, 50.

## 1.2 Supplementary Tables

**Supplementary Table 1. The list of primers used in this study.**

| **Name** | **Sequence** |
| --- | --- |
| Dpt-s | GCTGCGCAATCGCTTCTACT |
| Dpt-as | TGGTGGAGTGGGCTTCATG |
| AttA-s | GGCCCATGCCAATTTATTCA |
| AttA-as | AGCAAAGACCTTGGCATCCA |
| CecA1-s | ACGCGTTGGTCAGCACACT |
| CecA1-as | ACATTGGCGGCTTGTTGAG |
| RpL32-s | AGCATACAGGCCCAAGATCG |
| RpL32-as | AAACGCGGTTCTGCATGAG |
| Drs-s | ATGATGCAGATCAAGTAC |
| Drs-as | TCCCAGGACCACCAGCAT |
| Mtk-s | CAGTGCTGGCAGAGCCTCAT |
| Mtk-as | ATAAATTGGACCCGGTCTTG |
| PGRP-SC2-s | AACTACCTGAGCTACGCCGTGAT |
| PGRP-SC2-as | AGCAGAGGTGAGGGTGTTGGTATT |
| PGRP-SC2-5’-s | GAGCGTCGAGATCTCAGGAA |
| PGRP-SC2-5’-as | AAGCTTGTGCTTGTGGGTTT |
| PGRP-SC2-3’-s | GCTTCCTCGTCGACATTAGC |
| PGRP-SC2-3’-as | CCTCCAACACGGATGACAAC |

**Supplementary Table 2. The summary of Mi-2 interactome.**

| Candidate name | Fold change | Candidate name | Fold change |
| --- | --- | --- | --- |
| Prx2 | >100 | Lmpt | 6.09 |
| Df31 | >100 | Fas1 | 5.97 |
| Foxo | >100 | Dek | 5.29 |
| 14-3-3epsilon | >100 | CG16772 | 4.21 |
| CG3884-RB | >100 | CG13155 | 3.80 |
| Shv | >100 | RpS3 | 3.64 |
| CG17549 | >100 | CG43074 | 3.54 |
| Blw | >100 | Hsc70-3 | 3.43 |
| Syx1A | 17.69 | RpS19a | 3.23 |
| BomT2 | 14.75 | MetRS | 2.98 |
| SelR | 12.25 | Acbp3 | 2.81 |
| Ak1 | 8.17 | CanB | 2.34 |
| CG14757 | 8.05 | Cpr60D | 2.17 |
| Got2 | 6.17 | RpL | 2.09 |
